# Prevalence, drug resistance, molecular typing and comparative genomics analysis of MRSA strains from a tertiary A hospital in Shanxi Province, China

**DOI:** 10.3389/fmicb.2023.1273397

**Published:** 2023-09-22

**Authors:** Zhuru Hou, Benjin Xu, Ling Liu, Rongrong Yan, Jinjing Zhang, Jiaxin Yin, Peipei Li, Jianhong Wei

**Affiliations:** ^1^Department of Basic Medicine, Fenyang College of Shanxi Medical University, Fenyang, China; ^2^Key Laboratory of Lvliang for Clinical Molecular Diagnostics, Fenyang, China; ^3^Department of Medical Laboratory Science, Fenyang College of Shanxi Medical University, Fenyang, China; ^4^Department of Clinical Laboratory, Fenyang Hospital of Shanxi Province, Fenyang, China

**Keywords:** MRSA, resistance genes, virulence genes, molecular typing, comparative genomics

## Abstract

*Staphylococcus aureus* (*S. aureus*) is an important zoonotic pathogen that causes a high incidence rate and mortality worldwide. This study investigated the prevalence of methicillin-resistant *Staphylococcus aureus* (MRSA) strains in a tertiary A hospital in Shanxi Province, China, in order to determine the major epidemic clones as well as their antibiotic resistance and virulence characteristics. A total of 212 *S. aureus* strains were collected in this hospital, and were subjected to antimicrobial susceptibility testing, detection of virulence genes, resistance genes, and efflux pump genes. Among them, 38 MRSA strains were further subjected to detection of biofilm genes, assessment of biofilm formation ability, MLST, *spa* typing, SCC*mec* typing, and phylogenetic analysis. The majority of *S. aureus* strains came from the neonatology department, with secretions and purulent fluid being the main source of samples. The strains showed high resistance to penicillin (98.11%), erythromycin (64.62%) and clindamycin (59.91%), while being sensitive to vancomycin and linezolid. The detection rates of efflux pump genes and resistance genes were high, and there was a significant correlation between resistance gene types and phenotypes, with *mecA* showing a close correlation with oxacillin. The detection rates of virulence genes and the toxin gene profiles of MSSA and MRSA strains showed significant differences. And the detection rate of biofilm genes in MRSA strains was relatively high, with 13.16% of MRSA strains showing strong biofilm formation ability. The most common epidemic clone of MRSA was ST59-SCC*mec*IV-t437, followed by ST59-SCC*mec*V-t437. The former had a higher detection rate of resistance genes and a stronger biofilm formation ability, while the latter had a higher positive rate for *pvl* gene and stronger pathogenicity, making it more likely to cause systemic infections. Phylogenetic analysis showed that all MRSA strains in this study clustered into three major branches, with distinct differences in antibiotic resistance and virulence characteristics among the branches. ST59-MRSA strains from different species showed consistency and inter-species transmission, but there were differences among ST59-MRSA strains from different geographical locations. In general, most MSSA and MRSA strains exhibited multidrug resistance and carried multiple resistance genes, virulence genes, and biofilm formation genes, warranting further research to elucidate the mechanisms of drug resistance and pathogenesis.

## 1. Introduction

*Staphylococcus aureus* (*S. aureus*) is a widely distributed Gram-positive pathogenic bacterium that gets its name from the yellow grape-like colonies on culture media (Algammal et al., 2020b). It colonizes various parts of the human body, such as the nasal cavity, skin, throat, and digestive system, and is a major cause of endocarditis, bacteremia, osteomyelitis, and skin and soft tissue infections (Turner et al., 2019). The pathogenicity of *S. aureus* primarily stems from the production of exotoxins, including paton-valentine leukocidin (PVL), hemolysins, staphylococcal enterotoxins (SEs), adhesins, exfoliative toxins, etc., which enter the host’s body and cause disease through adhesion and invasion (Oliveira et al., 2018). SEs are the most common exotoxins and include *sea-see*, *seg-sej*, *sel-seq*, *ser-set*, *selk-selq*, *selu-selx*; they can disrupt intestinal functions and lead to food poisoning, often causing clinical symptoms of nausea, vomiting, abdominal pain, and diarrhea. PVL is mainly composed of two proteins encoded by the *lukS-PV* and *lukF-PV* genes, and it targets phagocytes, lymphocytes and natural killer cells, causing skin and soft tissue infections and severe necrotizing pneumonia (Bennett and Thomsen, 2020).

Due to the high pathogenicity of *S. aureus*, antibiotics have been widely used in clinical practice to treat *S. aureus* infections. However, just 2 years after the introduction of methicillin, methicillin-resistant *Staphylococcus aureus* (MRSA) emerged (Barber, 1961). Since then, MRSA strains have spread extensively in countries such as Europe and America, transitioning from hospitals to communities. MRSA has become an important pathogen causing both hospital-acquired and community-acquired infections and has evolved to some strains which develop resistance to virtually all clinically available antibiotics (Mlynarczyk-Bonikowska et al., 2022). MRSA is resistant to almost all β-lactam antibiotics, mainly due to the acquisition of a resistant genomic island called staphylococcal cassette chromosome (SCC) element carrying the *mecA/mecC* gene (SCC*mec*). The SCC*mec* element is a mobile genetic element that inserts into the chromosome of susceptible strains, resulting in the production of penicillin-binding protein (PBP2a/2c) and significantly reducing their affinity for β-lactam antibiotics, thus conferring resistance (Yamaguchi et al., 2020). Additionally, multidrug-resistant MRSA strains have acquired numerous resistance genes, which further enhance their resistance complexity and can be transmitted between different strains and species through mobile elements (Monaco et al., 2017).

As the understanding of the constantly changing epidemiology of MRSA increases, there is a need for rapid and reliable methods to characterize isolates to aid in investigating clone dissemination. The main methods for MRSA genotyping include pulsed field gel electrophoresis (PFGE), multilocus sequence typing (MLST), SCC*mec* typing, and *spa* typing. PFGE typing has high resolution, stable and reproducible results, and can effectively reflect the correlation between bacterial genotype and epidemiology. It is considered the “gold standard” for bacterial genotyping (He and Reed, 2020). MLST is based on the differences in seven housekeeping genes of *S. aureus*. This technique has high accuracy, good reproducibility, and allows typing results to be uploaded to public databases for data sharing and comparison, which is important for understanding strain epidemiological characteristics and genetic evolutionary relationships (Liu and Ji, 2020). SCC*mec* typing is based on the differences in *mec* gene complex and cassette chromosome recombinase (*ccr*) gene complex. It has been certified by the international working group on classification of SCC components and is divided into 14 types, SCC*mec* I - SCC*mec* XIV (Uehara, 2022). *spa* typing is a sequencing typing method based on the polymorphism of the X region sequence of *spa* gene, which encodes staphylococcal protein A. This method is fast, reproducible, and has high resolution, indicating both micro and macro variations, making it suitable for local and global epidemiological studies (Wang, 2020).

MRSA strains exhibit genetic diversity, and the main feature of their molecular epidemiology is the continuous emergence of regionally prevalent strains. The prevalence, drug resistance characteristics, virulence characteristics, and epidemic clones of MRSA vary in different regions and change over time. However, there were few reports on the molecular epidemiological characteristics of MRSA in Shanxi Province, China. Therefore, this study focused on 212 strains of *S. aureus* isolated from a tertiary A hospital in Shanxi Province. We investigated the colonization and departmental distribution of the strains, tested their resistance to commonly used clinical antibiotics, as well as the carriage rate of resistance genes, efflux pump genes and virulence genes. Molecular genetic methods such as SCC*mec* typing, MLST, *spa* typing, and whole-genome sequencing were used to genotype the MRSA isolates from different sources and perform comparative genomic analysis. This study provided initial insights into the prevalence characteristics, drug resistance, and genetic polymorphism of healthcare-associated MRSA in Shanxi Province, revealing the molecular features and evolutionary relationships of MRSA strains. It also provided a theoretical basis for the clinical treatment of MRSA infections and technical support for the tracking of healthcare-associated MRSA infections.

## 2. Materials and methods

### 2.1. Sample collection and strain isolation

From May 2021 to October 2022, a total of 212 non-duplicate strains of *S. aureus* were collected and isolated from a tertiary A hospital in Shanxi Province. *S. aureus* isolates were recovered from inpatients and outpatients who had cough, fever, skin abscess, and other clinical symptoms related to infection. These strains included all the isolates of *S. aureus* obtained from patients during this period at this hospital. The identification of the strains was performed using matrix-assisted laser desorption/ionization time-of-flight mass spectrometry (MALDI-TOF/TOF) technology. The resistance to oxacillin was determined through antibiotic susceptibility testing, and the presence of the *mecA* gene was confirmed by PCR, identifying 38 strains as MRSA. This study was approved by the Ethics Committee of Fenyang College, Shanxi Medical University. Being a retrospective study, informed consent was not required.

### 2.2. Antibiotic susceptibility testing

All strains were subjected to antimicrobial susceptibility testing according to the methods recommended by the Clinical and Laboratory Standards Institute (CLSI, 2015). The VITEK2-compact automated antimicrobial susceptibility analyzer was used to determine the minimum inhibitory concentrations of the strains against 12 antibiotics, including penicllin (10 U), erythromycin (15 μg), clindamycin (2 μg), oxacillin (1 μg), sulfamethoxazole (25 μg), tetracycline (30 μg), gentamicin (10 μg), levofloxacin (5 μg), rifampicin (5 μg), nitrofurantoin (300 μg), vancomycin (30 μg), linezolid (30 μg). ATCC29213 was used as the quality control strain.

### 2.3. Detection of resistance genes, virulence genes, and biofilm-related genes

PCR was performed to detect the positive rates of various genes, including 8 efflux pump genes (Zhang Hang et al., 2019), 17 resistance genes (Merlino et al., 2002; Chen Cheng et al., 2020), 22 virulence genes (Brakstad et al., 1992; Chen Cheng et al., 2020), and 11 biofilm-related genes (Waryah et al., 2016). The primers and PCR programs for amplification of the relevant genes were performed according to the primer sequences and PCR procedures listed in Supplementary Table S1. After the reaction, 6 × DNA loading buffer was added to the PCR products, and 20 μL of the mixture was loaded onto a 1.5% agarose gel for electrophoresis at 110 V for 40 min. The gel was visualized and images were captured using a UV gel imaging system. The positive rates of each gene were calculated.

### 2.4. Biofilm formation assay

100 μL of the MRSA strains, stored at −80°C, were added to 10 mL of TSB medium and incubated at 37°C with shaking at 220 rpm for 12 h. Then, 100 μL of the cultured bacterial suspension was added to 10 mL of 0.5% TSBG medium and mixed well. Next, 200 μL of the bacterial suspension was added to each well of a 96-well plate, with each strain occupying 5 wells. The plate was then incubated at 37°C for 24 h. After incubation, the liquid and planktonic bacteria were discarded by adding 200 μL of PBS buffer to each well and washing it three times. The wells were then stained with 100 μL of crystal violet dye and incubated for 30 min. After removing the dye, each well was washed three times with 200 μL of PBS buffer. Finally, 200 μL of 95% ethanol was added to each well, and the optical density at 560 nm was measured within 30 min. TSBG medium served as the negative control, and the critical optical density value was defined as ODc (mean OD of the negative control plus three times its standard deviation). The ability to form biofilms was classified into four categories: OD ≤ ODc, no biofilm formation (−); ODc<OD ≤ 2ODc, weak biofilm formation (+); 2ODc<OD ≤ 4ODc, moderate biofilm formation (++); 4ODc<OD, strong biofilm formation (+++).

### 2.5. Whole-genome sequencing and comparative genomics analysis

The genomes of a total of 38 MRSA strains were sequenced at the Beijing Genomics Institute (BGI, Shenzhen, China) using a PacBio Sequel II and DNBSEQ platform. The PacBio platform utilized four SMRT cells Zero-Mode Waveguide arrays for sequencing, and the resulting subreads set was generated. Subreads were repaired and the Canu program was employed for self-correction.

The assembled contigs were used for molecular typing, including MLST, *spa*-typing and SCC*mec* typing. These typings were predicted using the Center for Genomic Epidemiology website.^1^ For function annotation, the WGS data was annotated some databases, including VFDB (for virulence genes) (Chen et al., 2016), ARDB, CARD (for resistance genes) (Alcock et al., 2020) and COG (Galperin et al., 2015). Analyze the Core/Pan genes of all MRSA strains, as well as the COG functions of these genes. Cluster these strains based on Core/Pan genes, and construct the phylogenetic tree using the TreeBeST with the NJ method. Then 20 strains of ST59 MRSA strains were selected from NCBI, and these strains were compared with ST59 strains in this study to build a phylogenetic tree. The production and processing of images, such as heat maps, box plots, phylogenetic trees and so on, have been utilized on the chiplot online website.^2^

### 2.6. Statistical analyses

Statistical analyses were performed using Graphpad Pism 9.5 for Windows. Chi-squared test was used for rate analysis, and Pearson correlation test was used for consistency analysis. The *p* < 0.05 was considered to indicate statistical significance. *p ≤ 0.05; **p ≤ 0.01; ***p ≤ 0.001; ****p ≤ 0.0001.

## 3. Results

### 3.1. Sample source and department distribution of *Staphylococcus aureus*

A total of 38 out of the 212 identified *S. aureus* strains were confirmed to be MRSA (Table 1). These strains were sourced from various departments, with both methicillin-susceptible *S. aureus* (MSSA) and MRSA mainly originating from the neonatology department (31/174, 17.82%; 9/38, 23.68%). For MSSA, children (53/174, 30.46%), middle-aged individuals (59/174, 33.91%), and the elderly (54/174, 31.03%) were the predominant affected populations. On the other hand, MRSA primarily affected children (19/38, 50.00%) and the elderly (10/38, 26.32%). The main sources of the strains were secretions and purulent fluid, with 23 and 11 MRSA cases originating from secretions and purulent fluid, respectively. The remaining 4 cases were from sputum, throat swabs, vaginal swabs, and tissue samples. Statistical analysis showed significant differences in age and sample type, in addition to gender and department distribution. Detailed information on the MRSA strains can be found in Supplementary Table S2.

**Table 1:** The general information of *S. aureus* isolates.

		Group				*χ* ^2^	*p* value
		MSSA	%	MRSA	%		
Sex	Male	102	58.62%	23	60.53%	0.084	0.7721
	Female	72	41.38%	15	39.47%		
Age	≤9 year	53	30.46%	19	50.00%	8.767	0.0326
	9–18 year	8	4.60%	1	2.63%		
	18–50 year	59	33.91%	8	21.05%		
	>50 year	54	31.03%	10	26.32%		
Department	Neonatal pediatrics	31	17.82%	9	23.68%	12.689	0.1776
	Otolaryngology	24	13.79%	7	18.42%		
	Burn department	25	14.37%	3	7.89%		
	Pediatrics	10	5.75%	5	13.16%		
	Orthopedics	10	5.75%	3	7.89%		
	Endocrinology department	11	6.32%	2	5.26%		
	Obstetrics	11	6.32%	1	2.63%		
	General surgery department	7	4.02%	2	5.26%		
	Department of divine surgery	7	4.02%	1	2.63%		
	Other departments	38	21.84%	5	13.16%		
Specimen type	Secretion	116	66.67%	23	60.53%	22.034	0.0002
	Purulent fluid	19	10.92%	11	28.95%		
	Puncture fluid	14	8.05%	0	0		
	Blood	10	5.75%	0	0		
	Others	15	8.62%	4	10.53%		

### 3.2. The drug resistance characteristics of *Staphylococcus aureus* isolates

**Figure 1:**
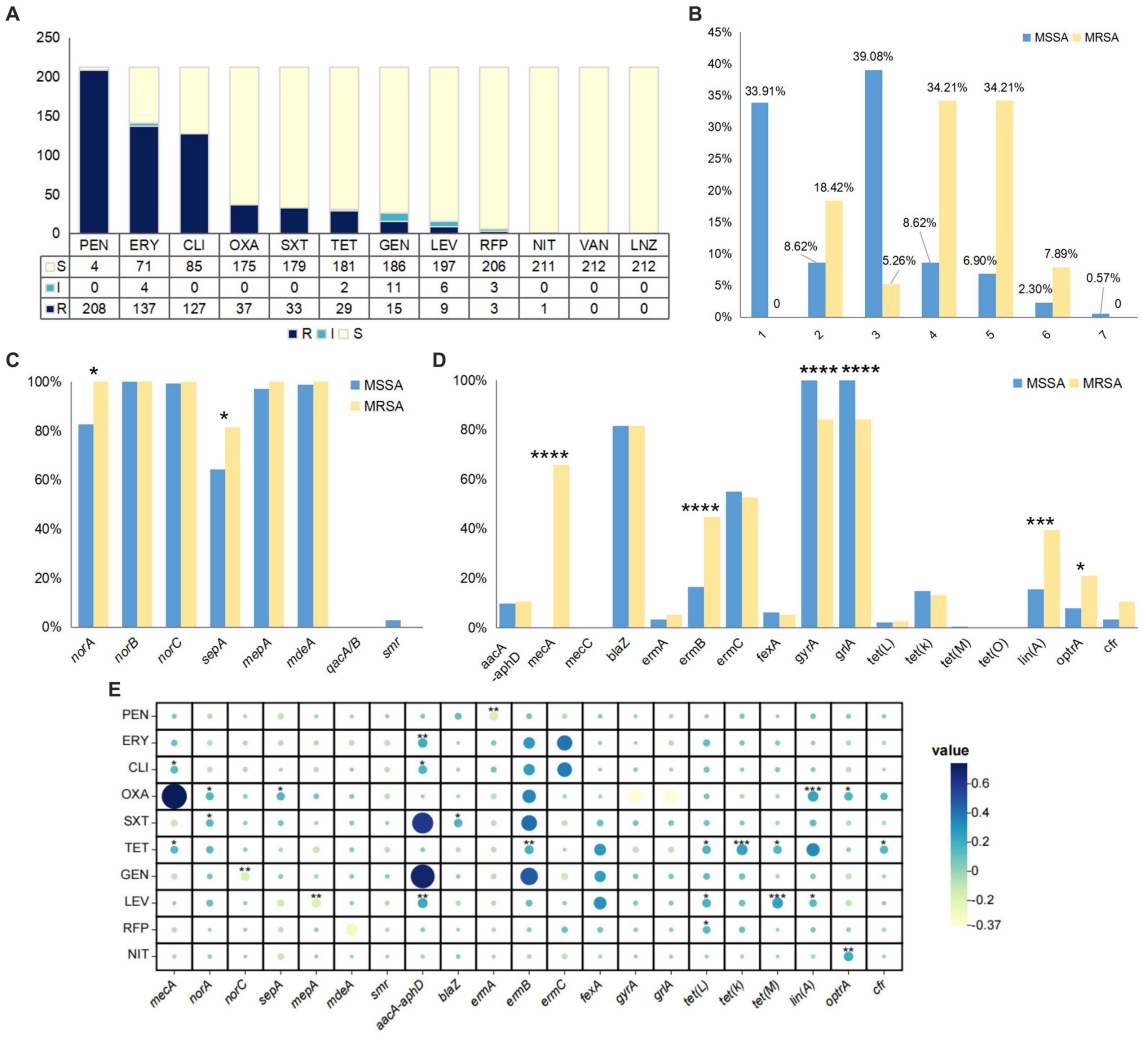
The drug resistance characteristics of *S. aureus* isolates. **(A)** The antibiotic susceptibility testing of all strains. PEN, penicillin; ERY, erythromycin; CLI, clindamycin; OXA, oxacillin; SXT, sulfamethoxazole; TET, tetracycline; GEN, gentamicin; LEV, levofloxacin; RFP, rifampicin; NIT, nitrofurantoin; VAN, vancomycin; LNZ, linezolid. **(B)** The number of antibiotic resistance of MSSA and MRSA. **(C)** Detection rate of efflux pump genes. **(D)** The detection rate of resistance genes. **(E)** Consistency between drug resistance genotypes and phenotypes. The size of the circle represents the correlation between the antibiotics and resistance genes, and the closer the correlation, the larger the circle. The *p* < 0.05 was considered to indicate statistical significance. *p ≤0.05; **p ≤0.01; ***p≤0.001; ****p≤0.0001.

In this study, *S. aureus* exhibited high resistance rates to penicillin (208/212, 98.11%), erythromycin (137/212, 64.62%), and clindamycin (127/212, 59.91%). It showed sensitivity to vancomycin and linezolid. Oxacillin, sulfamethoxazole, tetracycline, gentamicin, levofloxacin, rifampicin, and nitrofurantoin demonstrated good activity against *S. aureus*, with resistance rates ranging from 0.5 to 17% (Figure 1A). Multiple drug resistance was observed in 57.47% of the MSSA strains and 81.58% of the MRSA strains. One MSSA strain exhibited resistance to all seven antibiotics tested, while 26/38 MRSA strains demonstrated resistance to four or five antibiotics (Figure 1B). Detection of efflux pump genes revealed high detection rates for all genes except *qacA/B* and *smr*, which were not detected in MRSA strains. The detection rates for *norA*, *norB*, *norC*, *mepA*, and *mdeA* genes in MRSA strains were 100%, slightly higher than their detection rates in MSSA strains (Figure 1C). The most common efflux pump gene profile in all strains was *norA-norB-norC-sepA-mepA-mdeA* (120/212, 56.60%). Detection of resistance genes showed that among the 37 oxacillin-resistant strains, 24 were *mecA-*positive, while one strain was sensitive to oxacillin but tested positive for the *mecA* gene. In all strains, quinolone-related genes *gyrA* and *grlA* (174/174, 100%; 32/38, 84.21%), and β-lactam-related gene *blaZ* (142/174, 81.61%; 31/38, 81.58%) exhibited the highest detection rates. But *tet(O)* and *mecC* were not detected in any of the strains (Figure 1D). The most common resistance gene profile in all strains was *blaZ-ermC-gyrA-grlA* (51/212, 24.06%). Consistency analysis of all efflux pump genes, resistance genes, and antibiotic detection results revealed a significant correlation between resistance genotypes and phenotypes, showing a positive correlation. When the Pearson correlation coefficient is greater than 0.4, it indicated a strong correlation. The closely related correlations found in this study were *mecA* and oxacillin (*r* = 0.7378), *aacA-aphD* and gentamicin (*r* = 0.6943), *aacA-aphD* and sulfamethoxazole (*r* = 0.598), *ermB* and gentamicin (*r* = 0.4772), *ermB* and sulfamethoxazole (*r* = 0.4127) (Figure 1E).

### 3.3. The virulence characteristics of *Staphylococcus aureus* isolates

**Figure 2:**
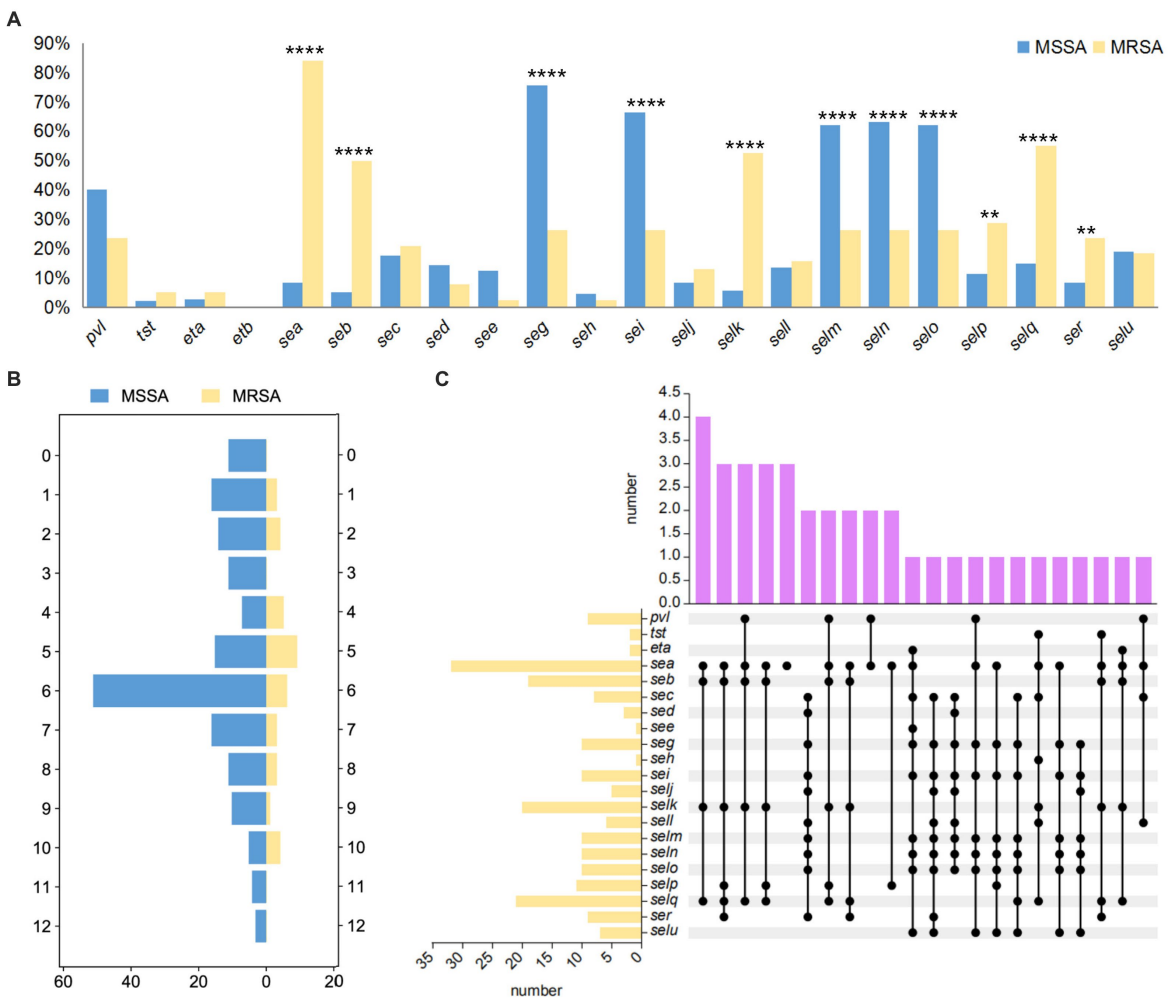
The virulence characteristics of *S. aureus* isolates. **(A)** The detection rate of virulence genes of all strains. **(B)** The number of virulence genes of MSSA and MRSA. The horizontal axis represents the number of strains, while the vertical axis represents the number of genes. **(C)** The upset plot of MRSA. The bar chart on the left represents the quantity of various toxic genes. The solid circles represent the intersection between data quantities, meaning that each vertical column of solid circles represents a specific toxic gene profile. The bar chart above represents the quantity of different toxic gene profiles. The *p* < 0.05 was considered to indicate statistical significance. **p ≤0.01; ****p≤0.0001.

In this study, all enterotoxin genes were detected, but the detection rates varied significantly among different strains. Among MSSA strains, *seg* had the highest detection rate (132/174, 75.86%), followed by *sei* (116/174, 66.67%), *seln* (110/174, 63.22%), *selo* (108/174, 62.07%), and *selm* (108/174, 62.07%). In MRSA strains, *sea* had the highest detection rate (32/38, 84.21%), followed by *selq* (21/38, 55.26%), *selk* (20/38, 52.63%), and *seb* (19/38, 50.00%). The detection rate of the *pvl* gene was relatively high, and the detection rate in MSSA strains (70/174, 40.23%) was higher than that in MRSA strains (9/38, 23.68%). The detection rates of toxic shock syndrome toxin (*tst*) gene and exfoliative toxin (*eta*) gene were maintained at 2 to 5.5%, while the exfoliative toxin (*etb*) gene was not detected in any strains (Figure 2A). The most common virulence gene profile in all strains was *pvl-seg-sei-selm-seln-selo* (43/212, 20.28%). The distribution of virulence gene numbers in MSSA strains ranged from 0 to 12, with 29.31% of strains harboring 6 virulence genes. Low-pathogenicity strains without virulence genes accounted for 6.32% of MSSA strains, while 1.72% of strains had 12 virulence genes and exhibited high pathogenicity. Considering the clinical information, all highly pathogenic strains were derived from the otolaryngology department. MRSA strains mainly had 4 to 6 or 10 virulence genes, generally demonstrating strong pathogenicity (Figure 2B). Figure 2C analyzed the virulence gene profiles of the 38 MRSA strains and showed a complex pattern, with a total of 22 types, among which *sea-seb-selk-selq* were the most common.

### 3.4. Biofilm detection of MRSA

The positive rates of biofilm-associated genes in the 38 MRSA strains were analyzed in Supplementary Figure S1A, and except for *fib*, which was not detected, the positive rates of the remaining genes were all above 80%. Among them, *clfA*, *clfB*, *fnbA*, *icaA*, *icaB*, *icaC*, and *icaR* were detected in all MRSA strains. The biofilm formation ability test results showed that, except for strains SA17 and SA35, all other strains were capable of biofilm formation (Supplementary Figure S1B). Most strains showed weak (17/38, 44.74%) or moderate (14/38, 36.84%) biofilm formation ability, while only 5 strains showed strong biofilm formation ability (Supplementary Figure S1C).

### 3.5. Molecular typing of MRSA

**Figure 3:**
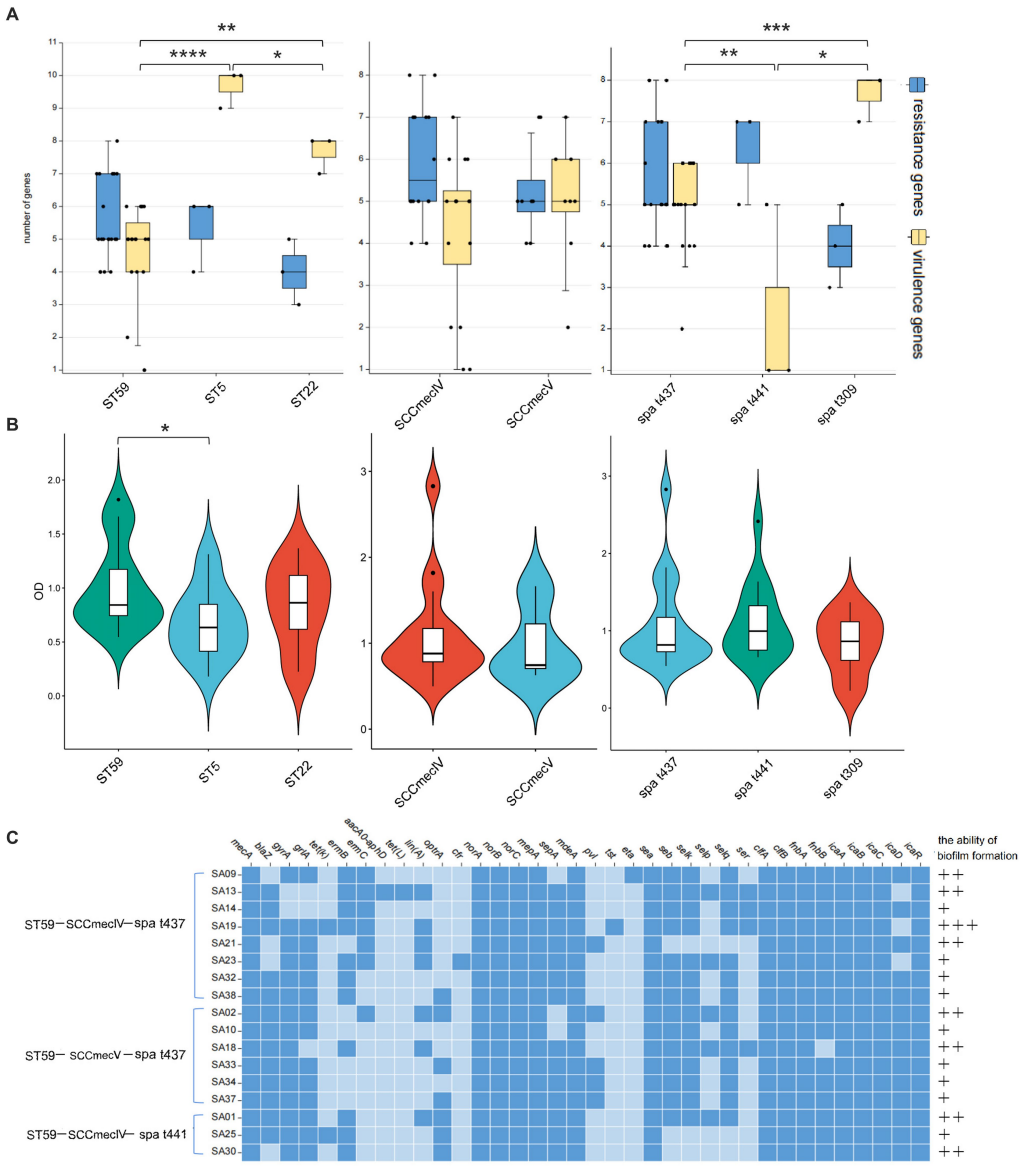
The resistance and virulence characteristics of major typings. **(A)** The number of resistant and virulence genes in the main typing strains. The horizontal axis represents different subtypes, while the vertical axis represents the number of genes. The box plot represents the average, upper and lower quartiles of these gene numbers. **(B)** The biofilm formation ability in the main typing strains. The horizontal axis represents different subtypes, while the vertical axis represents OD value. The violin diagram shows the distribution and probability density of biofilm formation ability. **(C)** The gene heatmap of major epidemic typings. Dark blue indicates the presence of this gene, while light blue indicates the absence of this gene. The formation ability of biofilms varies from strong (+++) to weak (+). The *p* < 0.05 was considered to indicate statistical significance. *p ≤0.05; **p ≤0.01; ***p≤0.001; ****p≤0.0001.

A total of 14 MLST types were identified among the 38 MRSA strains, with ST59 being the most prevalent type (19/38, 50.00%), followed by ST22 (3/38, 7.80%) and ST5 (3/38, 7.80%). SCC*mec* typing revealed the presence of three major types and five minor subtypes, including IVa (14/25, 56%), IVc (1/25, 4%), IVg (1/25, 4%), Vb (8/25, 32%), and XII (1/25, 4%). Additionally, 13 MRSA strains were detected to be *mecA*-negative and SCC*mec* element-negative, but still exhibited resistance to oxacillin. In terms of *spa* typing, except for strain SA27, the remaining 37 MRSA strains were classified into 15 types, with t437 being the most prevalent (17/37, 45.95%), followed by t441 (3/37, 8.11%) and t309 (3/37, 8.11%) (Supplementary Table S2). MLST typing, SCC*mec* typing, and *spa* typing showed a close relationship, with all ST22 types corresponding to t309, while ST59 types were predominantly associated with SCC*mec*-IV, V and *spa* t437 and t441. The most common epidemic clone in this study was ST59-SCC*mec* IV-t437 (8/38, 21.05%), followed by ST59-SCC*mec* V-t437 (6/38, 15.79%) and ST59-SCC*mec* IV-t441 (3/38, 7.89%). Analysis of the number of resistance genes and virulence genes in the major types showed that ST59 had a higher number of resistance genes but fewer virulence genes compared to ST5 and ST22. There was no significant difference in the number of resistance genes and virulence genes between SCC*mec* IV and V. The *spa* type t437 exhibited high levels of resistance genes and virulence genes, t441 had more resistance genes but very few virulence genes, and t309 showed the opposite pattern (Figure 3A). Different strains of different molecular types exhibited varying biofilm formation abilities, with ST59-MRSA strains showing higher biofilm formation abilities, and significant differences were observed between ST59-MRSA and ST5-MRSA strains (Figure 3B). Figure 3C analyzed the resistance gene profiles, virulence gene profiles, and biofilm formation abilities of the three major epidemic clones. ST59-SCC*mec* V-t437 had a high positive rate for the *pvl* gene, reaching 66.67%. The *ser* gene rate for the ST59-SCC*mec* IV-t437 type was relatively high at 37.5%, and this type of MRSA strain showed higher biofilm formation abilities.

### 3.6. Phylogenetic analysis

**Figure 4:**
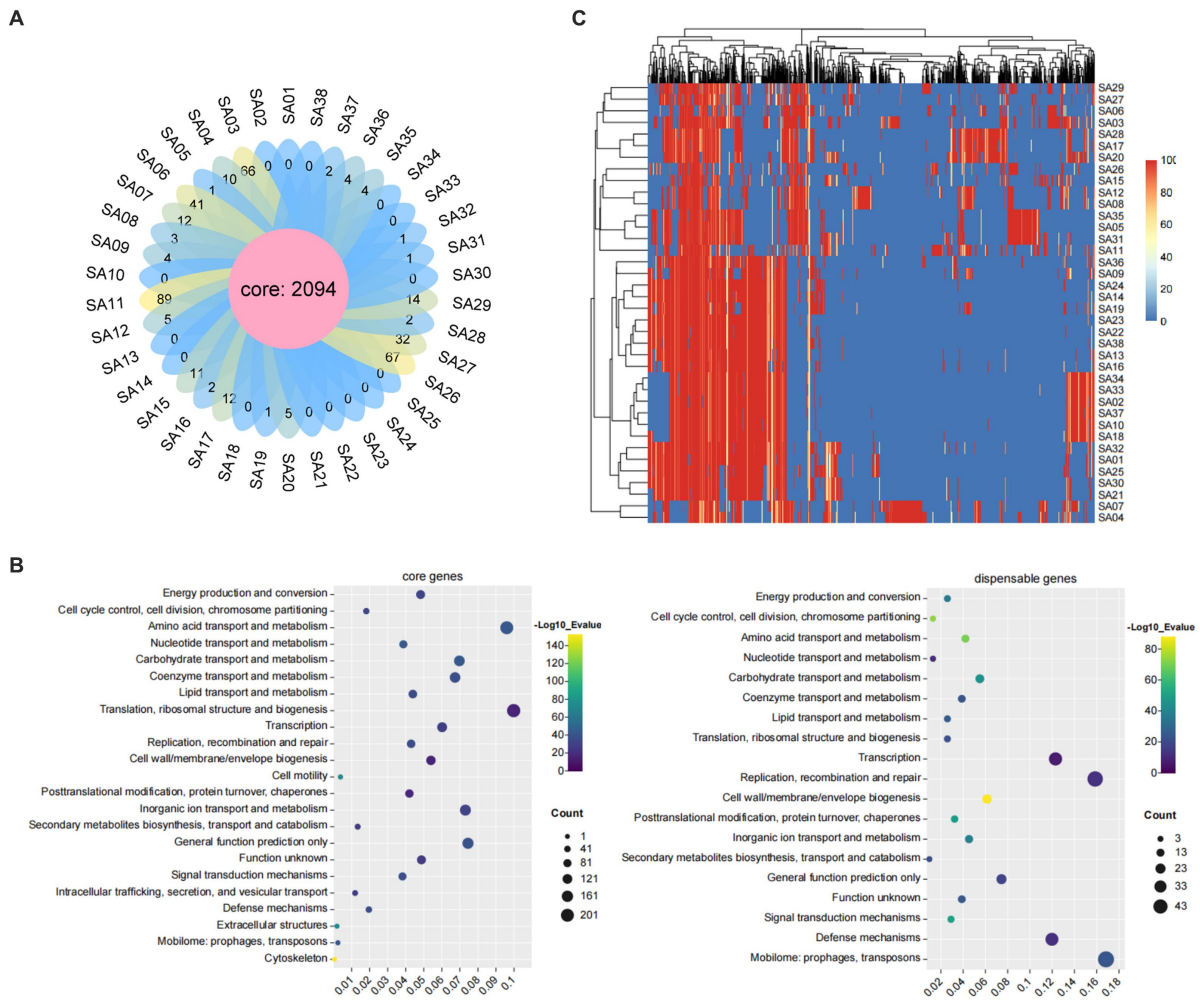
The core and pan-genome analyses of 38 MRSA in this study. **(A)** The flower diagram of numbers of core genes and dispensable genes for all the strains. **(B)** The COG functional annotation of core genes and dispensable genes. **(C)** The heat map of dispensable genes. Top are Dispensable gene cluster and left are strain cluster. The similarities of gene are shown in the middle with different color represent different coverage by heat map.

Core-pan analysis of the 38 MRSA strains revealed a total of 2094 core genes and 0–66 dispensable genes distributed among the strains, with SA03, SA11, and SA26 having the highest number of dispensable genes (Figure 4A). COG functional annotation analysis was performed on the 2094 core genes and 309 dispensable genes, resulting in annotations to 23 and 19 categories, respectively. The core genes were involved in a wide range of functions, primarily in translation, ribosomal structure, and biogenesis (9.98%), followed by amino acid transport and metabolism (9.60%). The dispensable genes were primarily involved in mobilome: prophages, transposons (16.83%), and replication, recombination, and repair (15.86%) (Figure 4B). A heatmap was created based on the distribution of dispensable genes among different strains, which also showed clustering of the strains. The results revealed that these 38 strains mainly clustered into four major branches, with SA04 and SA07 forming a separate branch that was more distantly related to the other strains (Figure 4C).

**Figure 5:**
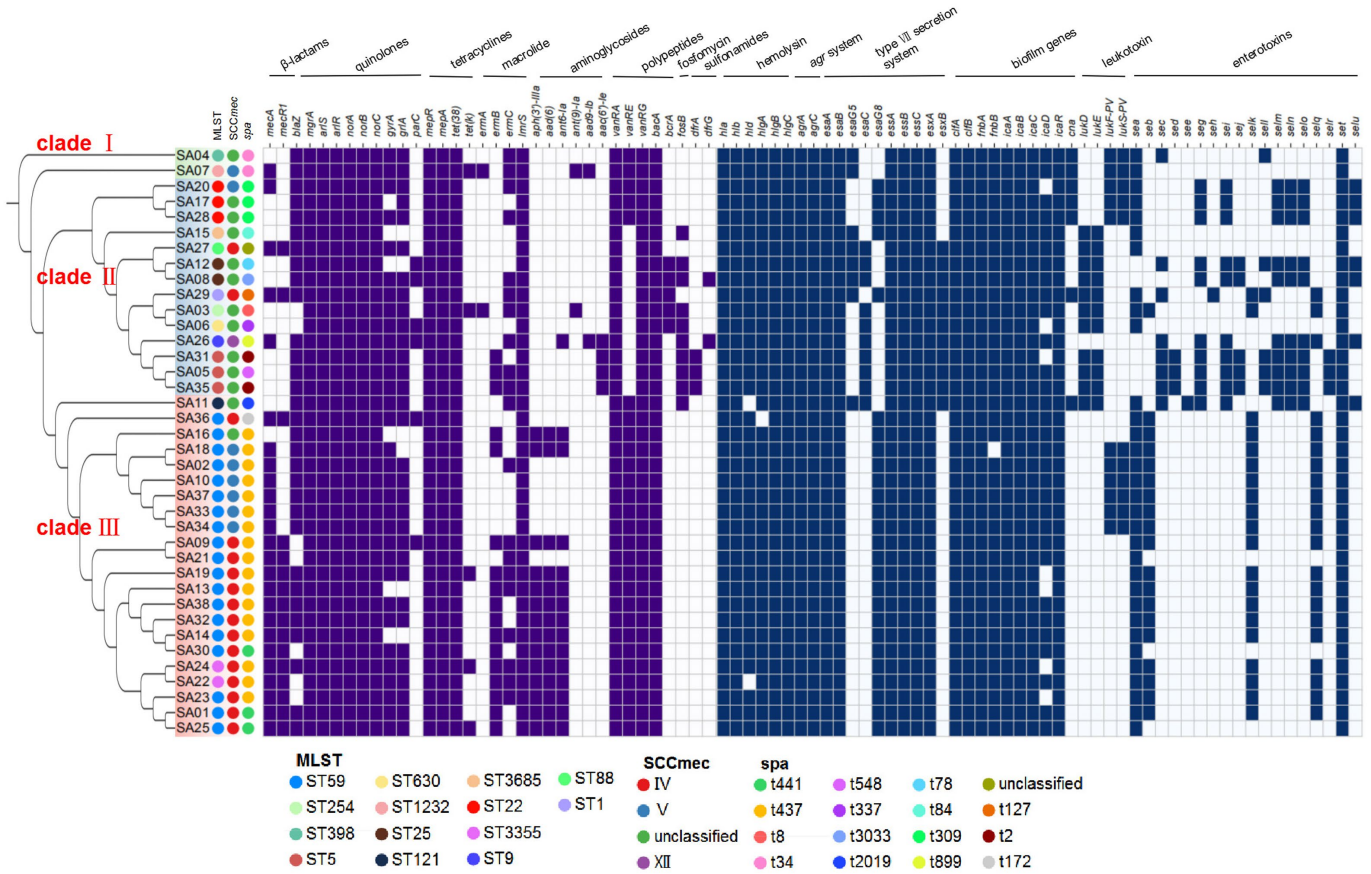
The phylogenetic trees of 38 MRSA strains. Perform phylogenetic analysis on 38 MRSA strains and construct a phylogenetic tree. These strains were divided into three branches, namely green (clade I), blue (clade II), and red (clade III). The different colors of the circles represented the different subtypes of the strains. Blocks indicated the presence or absence of these genes, dark blue indicated the presence of these genes, and light blue indicated the absence of these genes.

According to the analysis of core-pan genes, a phylogenetic tree of the 38 strains was constructed (Figure 5). The results showed that these strains were closely related and mainly clustered into three major branches, clade I, clade II, and clade III. SA04 and SA07 formed a separate branch, clade I. Clade II mainly consisted of strains that were not classified in SCC*mec* typing, and these strains were resistant to oxacillin but did not contain the *mecA* gene. Additionally, the positive rate of the resistance gene *blaZ*, associated with β-lactam antibiotics, was higher than the average level, reaching 85.7% in clade II. Clade III mainly consisted of MRSA strains of ST59 genotype, which exhibited significant differences in resistance and virulence genes compared to clade II. In clade III, the *vanRE* gene had a high detection rate of 100%, while *aph(3′)-IIIa*, *aad(6)*, and *ant6-Ia* had detection rates of 63.6%. In clade II, the detection rates of *vanRE* and *ant6-Ia* were 21.4 and 7.1% respectively, and *aph(3′)-IIIa* and *aad(6)* were not detected. The sulfonamide resistance genes *dfrA* and *dfrG* were not detected in clade III, while they had detection rates of 21.4% in clade II. The *lukD/lukE* gene, associated with leukocidin, had a high detection rate of 71.4% in clade II, while it was 4.5% in clade III. The *lukF-PV/lukS-PV* gene had similar detection rates in clade III and clade II, at 27.2 and 21.4%, respectively. Clade II and clade III had different profiles of common enterotoxin genes, which were *seg-sei-selm-seln-selo-set* and *sea-seb-selk-selq-set*, respectively.

### 3.7. The evolutionary analysis of ST59-MRSA

**Figure 6:**
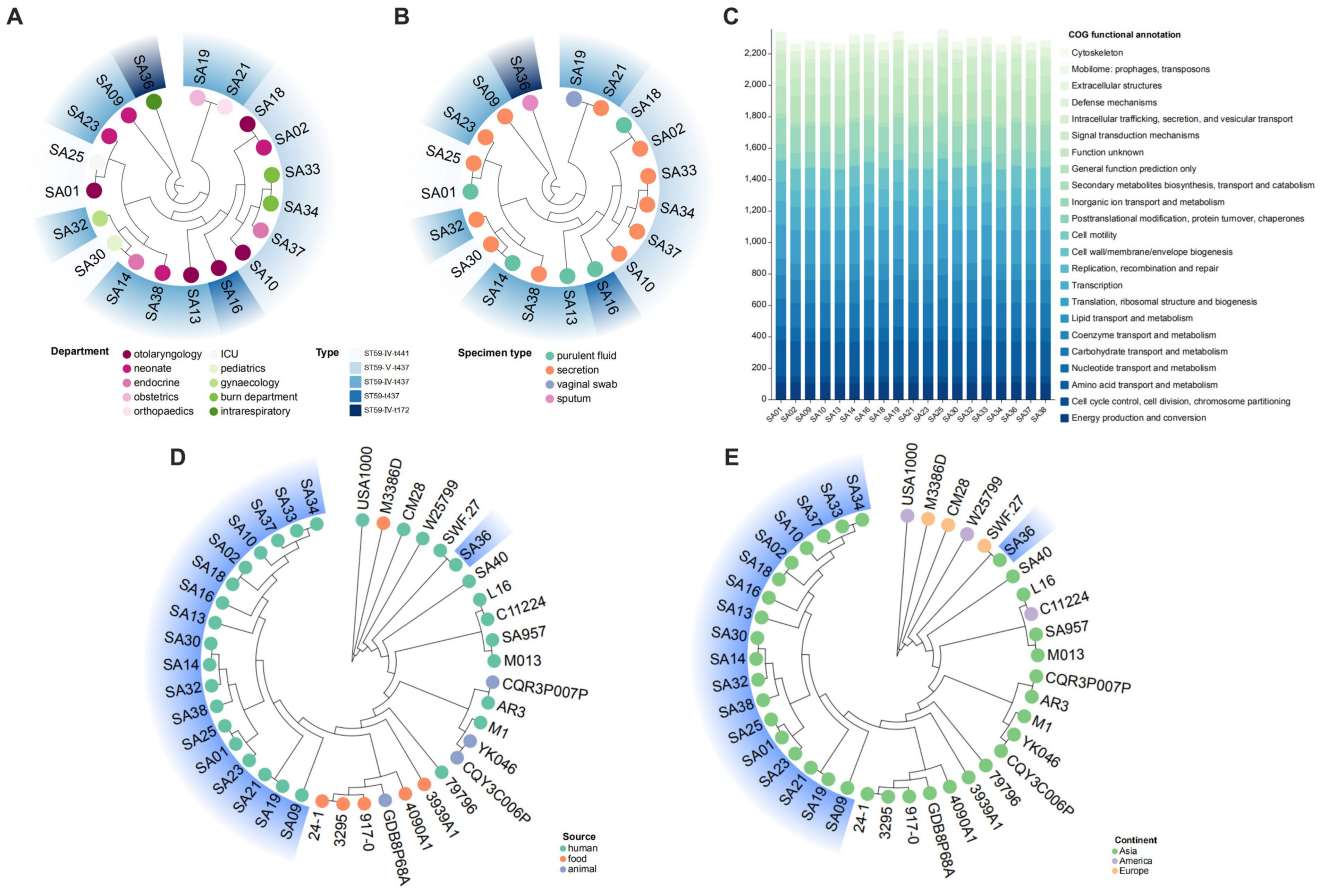
The evolutionary analysis of ST59 typing. **(A,B)** The phylogenetic trees of ST59 MRSA strains in this study. Analyze the impact of differences in departments **(A)** and speciman types **(B)** on the genetic relationships of strains. **(C)** The COG functional annotation of ST59 MRSA strains in this study. **(D,E)** The phylogenetic trees of ST59 MRSA strains in this study and 20 reference strains. Analyze the impact of differences in sources **(D)** and continents **(E)** on the genetic evolution of strains.

In this study, ST59-MRSA was the most common clone type, and a phylogenetic tree of 19 ST59-MRSA strains was constructed based on core-pan genes (Figures 6A,B). The phylogenetic tree showed that these strains clustered into three branches, indicating diversity. Strains of the ST59-SCC*mec* V-t437 type were closely related and formed a single branch. Figures 6A,B showed that differences in departments and sample types did not have a significant impact on the phylogenetic relationship of strains. Strains from different departments exhibited similarities, while strains from the same department exhibited diversity. COG functional annotation was performed on the 19 ST59-MRSA strains, and they were annotated to 23 entries, showing some variation between the genomes, but primarily annotated to amino acid transport and metabolism and translation, ribosomal structure, and biogenesis (Figure 6C). To study the genetic evolutionary relationship of ST59-MRSA, 22 ST59-MRSA strains were selected from the NCBI database as reference sequences (Supplementary Table S3), and a phylogenetic tree was constructed with them and the 19 ST59-MRSA strains from this study (Figures 6D,E). Except for SA36, the remaining 18 MRSA strains from this study clustered into the same branch. SA36 had the closest relationship with strain SWF.27 from European human sources, both belonging to ST59-t172. Figure 6D showed that ST59-MRSA strains from humans, animals, and food sources were closely related and exhibited consistent relationships. Figure 6E showed that the phylogenetic relationship between strains was related to geographic location, with ST59-MRSA primarily originating from Asia and exhibiting distinct regional characteristics. ST59-MRSA strains from the Americas and Europe tended to be in different evolutionary branches compared to those from Asia.

## 4. Discussion

MRSA is characterized by its wide spread, fast transmission, and complex resistance mechanisms, making the monitoring and treatment of MRSA an important topic of concern. According to the surveillance by the CHINET surveillance system, *S. aureus* has maintained a high detection rate, while the infection rate of MRSA decreased from 45 to 30% between 2013 and 2021. In some Asian countries, the hospital-acquired MRSA infection rates remain above 20%, with rates reaching as high as 70–80% in countries like South Korea and Vietnam (Chen and Huang, 2014). In our study, the MRSA infection rate was relatively low at 17.9%, below the national average. Children and elderly patients were the most common groups affected by MRSA infection, and there was a significant difference in MRSA infection rates based on age. Previous research has shown a decline in the outpatient visit rate for pyogenic skin and soft tissue infections in pediatrics, but *S. aureus* remains a major cause of invasive bacterial infections in children (Prochaska et al., 2023).

Antibiotic susceptibility testing results showed that most strains exhibited high resistance to penicillin, erythromycin, and clindamycin, while showing good antibacterial activity against vancomycin and linezolid, which is consistent with a multicenter longitudinal study of MRSA strains in China conducted by Wang B. et al. (2022). However, in this study, the resistance rates to tetracycline and gentamicin were 13.68 and 7.08%, respectively, significantly lower than the national average (42.8, 33.5%), which may be due to the lower usage rate of tetracycline and gentamicin in the Shanxi hospital. The multidrug resistance of *S. aureus* is not only present in clinical strains but also in retail water products in China. Monitoring of *S. aureus* in these products found that 90.6% of strains exhibited multidrug resistance, with high resistance against penicillin (88.2%), ampicillin (88.2%), and erythromycin (53.8%) (Rong et al., 2017). In this study, in addition to 81.58% of MRSA strains, 57.47% of MSSA strains also exhibited multidrug resistance, which was associated with the high detection rate of resistance genes and efflux pump genes. The resistance of beta-lactam antibiotics is mainly related to *mecA* and *blaZ*, with a high detection rate of *blaZ* in all strains. It encodes β-lactamase, which mediates the degradation of β-lactam antibiotics (Rocha et al., 2022). We also found 34.21% of MRSA strains showed resistance to oxacillin but were negative for the *mecA* and *mecC* genes. This may be due to the overproduction of β-lactamase or modifications in PBP genes caused by spontaneous amino acid substitutions in the transpeptidase domain (Hou et al., 2023). The detection rate of *pvl* gene in this type of MRSA was only 15.3%, which confirms that this type of MRSA generally does not contain the PVL locus that expresses leukocidin. Resistance to erythromycin is mainly determined by the resistance gene *erm*. Consistent with previous research, the highest positive rates of *ermC* (52.63%) and *ermB* (44.74%) were observed in MRSA strains, with *ermC* playing a dominant role in the mechanism of erythromycin resistance (Li et al., 2019). And also, *erm* can mediate the resistance of *S. aureus* to most macrolides, lincosamides, and streptogramins B (MLS-B), that is the *ermA* or *ermC* genes often determine the inducible resistance to MLS-B in *S. aureus*. The frequency of formation of constitutive variants from inducible *ermA* and *ermC* genes are usually high. The reasons behind constitutive variants include deletions, duplications, insertions, and, to a lesser extent, point mutations found in a region approximately 200 bp upstream of the 5′ end of the *erm* gene (Mlynarczyk-Bonikowska et al., 2022). But in this study, three point mutations (T222C, C224T, G299A) occurred in the *ermB* gene of SA05 and SA09 strains, resulting in ones synonymous mutation (Asn→Asn) and two missense mutations (Thr→Ile, Ser→Asn). However, these mutations do not affect the resistance of both strains to erythromycin and clindamycin. Pearson correlation analysis showed consistency between the resistance genotypes and phenotypes of *S. aureus*, and the use of conserved genes in *S. aureus* was able to successfully predict the resistance phenotype of strains. This suggests that a large hospital can use genomic data features to construct strain prediction models, which can be directly used to determine the drug resistance of strains and guide treatment in the absence of rapid susceptibility results (Wang S. et al., 2022). Therefore, the results of the study on the resistance genotypes and phenotypes of these *S. aureus* strains can serve as a medication guide for the treatment of *S. aureus* infections in this hospital and assist in the identification and treatment of multidrug-resistant bacteria in clinical practice.

In this study, all isolates contained at least one virulence gene, which plays an important role in colonization, invasion, and infection processes (Patel and Rawat, 2023). Enterotoxin is one of the main causes of foodborne illnesses caused by *S. aureus*. There were significant differences in the enterotoxin gene profiles between MSSA and MRSA strains. The classical enterotoxin genes *sea* (84.21%) and *seb* (50.00%) were more frequently detected in MRSA strains. In a study on subclinical mastitis in cows and water buffaloes, milk samples were collected, and MRSA isolates were tested for toxin genes, with the *sea* gene being the most prevalent (Algammal et al., 2020a). The *sea* gene is heat-stable and can maintain some activity under conditions of 28°C for 121 min. It is commonly detected in foodborne MRSA strains and has a significant association with outbreaks of food poisoning (Lv et al., 2021). The *seb* gene is usually highly expressed in ST59-MRSA, which contributes to widespread systemic infection, which may be the reason for the increased mortality rate of ST59 infection in China (Bae et al., 2021). In MSSA strains, the most common enterotoxin gene profile is *seg-sei-selm-seln-selo*, which belongs to the enterotoxin gene cluster (*egc* cluster). The *egc* cluster functions as a superantigen, activating T lymphocytes and antigen-presenting cells to produce a large amount of cytokines, which can directly cause toxicity to the cardiovascular system (Patel and Rawat, 2023). The *egc* cluster has become a major enterotoxin gene in *S. aureus*, with similar findings in oral *S. aureus* (Kwapisz et al., 2020) and foodborne *S. aureus* (Wu et al., 2019; Igbinosa et al., 2023). We found that the distribution of enterotoxin genes in this hospital is diverse, with different toxin gene distributions in different populations and disease types. Classical enterotoxin genes and *egc* clusters have become the main toxin genes in healthcare-associated and foodborne *S. aureus*. PVL is a two-component toxin composed of LukS and LukF proteins, which bind to C5aR and C5L2, forming channels on the cell membrane of white blood cells, leading to cell lysis and tissue necrosis, causing skin and soft tissue infections (Patel and Rawat, 2023). The detection rate of the *pvl* gene in this study was relatively high at 37.26%, higher than the detection rate of the *pvl* gene isolated from blood samples by Wang et al. (2021). PVL is commonly found in community-acquired methicillin-resistant *Staphylococcus aureus* (CA-MRSA) and has been considered a marker for CA-MRSA (Hou et al., 2023). Therefore, based on the detection rate of *pvl* and preliminary epidemiological investigations indicating that these strains in this study are mostly community-acquired infections, it suggests that CA-MRSA is not only spreading in the community but also increasing in prevalence in hospitals. In addition, the detection rate of *pvl* in MSSA strains (70/174, 40.23%) was higher than that in MRSA strains (9/38, 23.68%), which may be related to the source and number of samples. However, it also indicates that although MRSA has attracted attention due to its multidrug resistance, it is not necessarily more virulent than MSSA.

The clone types of *S. aureus* have geographical advantages, and ST59 is the predominant MRSA clone in China and even Asia (Huh and Chung, 2016). In this study, 50% of the MRSA isolates belonged to the ST59-MRSA. Systematic evolutionary analysis of ST59-MRSA showed consistency among strains isolated from humans, food, and animals, indicating close genetic relatedness. Additionally, the phylogenetic relationship among strains was associated with geographical location, with strains from different continents tending to diverge in evolution. In a study by Wang B. et al. (2022) in 2022, ST59-MRSA accounted for 27.1% of the clinical MRSA isolates in China. Wu et al. (2019) analyzed food-related MRSA in China in 2019 and found that 47.7% of the isolates belonged to ST59-MRSA, and it has been shown that food-related MRSA strains have a high degree of similarity to community-associated MRSA. The distribution of ST59-MRSA is geographically limited and mainly spreads within Asia. However, analysis of isolates from Europe showed that the resistance profiles of European ST59-t437 isolates were similar to those of Asian ST59-t437 isolates, but different from other MRSA isolates in Europe (Glasner et al., 2015).

In this study, the predominant epidemic clones were ST59-SCC*mec*IV-t437 and ST59-SCC*mec*V-t437, both belonging to clade III in the phylogenetic tree but relatively independent, forming two smaller branches. Both clones showed high rates of resistance and virulence gene detection, with resistance genes detected for all antibiotic classes except sulfonamides. The main enterotoxin genes in these clones were the classical enterotoxin genes *sea* and *seb*, and the ST59-SCC*mec*V-t437 clone had a high positive rate for the *pvl* gene, reaching 66.67%. These two clones belong to the Asian-Pacific clone and the Taiwan clone, respectively. Asian-Pacific clone isolates are mostly *pvl*-negative and colonize healthy children, while Taiwan clone carries the *pvl* gene and is generally isolated from critically ill patients. Due to the high expression of *pvl* and *hla* genes in Taiwan clone isolates, it has higher virulence and pathogenic potential compared to the Asian-Pacific clone (Chen et al., 2013). MLST is consistent with *spa* typing, and the ST59 typing is generally associated with t441 and t437. And then we found an ST59-t172 isolate, which is distantly related to other ST59-MRSA strains in this study but closely related to an ST59-t172 isolate from Finland in Europe. It is extremely rare elsewhere and mainly reported in Finland. The ST59-t172 strain mainly spreads among elderly patients and can cause outbreaks of *S. aureus* in environments with low MRSA incidence (Silvola et al., 2022).

## 5. Conclusion

This study investigated the colonization and distribution of *S. aureus* in a tertiary A hospital in Shanxi, China. It detected the antibiotic resistance, biofilm formation capability, as well as the carriage rates of resistance genes, virulence genes, and biofilm-related genes in the isolates. And then three molecular typing methods and whole-genome sequencing were used to study the molecular characteristics and genetic evolution of MRSA isolates from different sources. It was found that MRSA isolates displayed multidrug resistance, and the carriage of resistance genes and virulence genes exhibited diversity and complexity. The predominant clones were ST59-SCC*mec*IV-t437 and ST59-SCC*mec*V-t437, and ST59-MRSA was closely associated with various hosts, showing diverse patterns of transmission. This study elucidated the molecular characteristics and evolutionary relationship of MRSA isolates, which can contribute to the prevention and control of *S. aureus* infections.

## Data availability statement

The datasets presented in this study can be found in online repositories. The names of the repository/repositories and accession number(s) can be found at: https://www.ncbi.nlm.nih.gov/, PRJNA994481.

## Ethics statement

The studies involving humans were approved by Scientific Research Ethics Committee, Fenyang College of Shanxi Medical University. The studies were conducted in accordance with the local legislation and institutional requirements. Written informed consent for participation was not required from the participants or the participants’ legal guardians/next of kin in accordance with the national legislation and institutional requirements.

## Author contributions

ZH: Conceptualization, Data curation, Investigation, Methodology, Visualization, Writing – original draft, Writing – review & editing. BX: Conceptualization, Data curation, Funding acquisition, Investigation, Methodology, Project administration, Supervision, Visualization, Writing – original draft, Writing – review & editing. LL: Conceptualization, Funding acquisition, Investigation, Project administration, Visualization, Writing – original draft, Writing – review & editing. RY: Writing – review & editing, Investigation, Methodology, Resources, Validation. JZ: Writing – review & editing, Investigation, Methodology, Resources, Validation. JY: Writing – review & editing, Data curation, Investigation, Methodology. PL: Writing – review & editing, Data curation, Investigation, Methodology. JW: Validation, Writing – review & editing, Resources, Software, Supervision.

## Footnotes

^1^ https://cge.cbs.dtu.dk/services

^2^ https://www.chiplot.online/
