# Supplementary material for: Prevalence, drug resistance, molecular typing and comparative genomics analysis of MRSA strains from a tertiary A hospital in Shanxi Province, China
Source: Front Microbiol. 2023 Sep 22;14:1273397. doi: 10.3389/fmicb.2023.1273397 (PMC10556501; doi:10.3389/fmicb.2023.1273397)
Supplement: Supplementary file 1 [file Data_Sheet_1.docx]

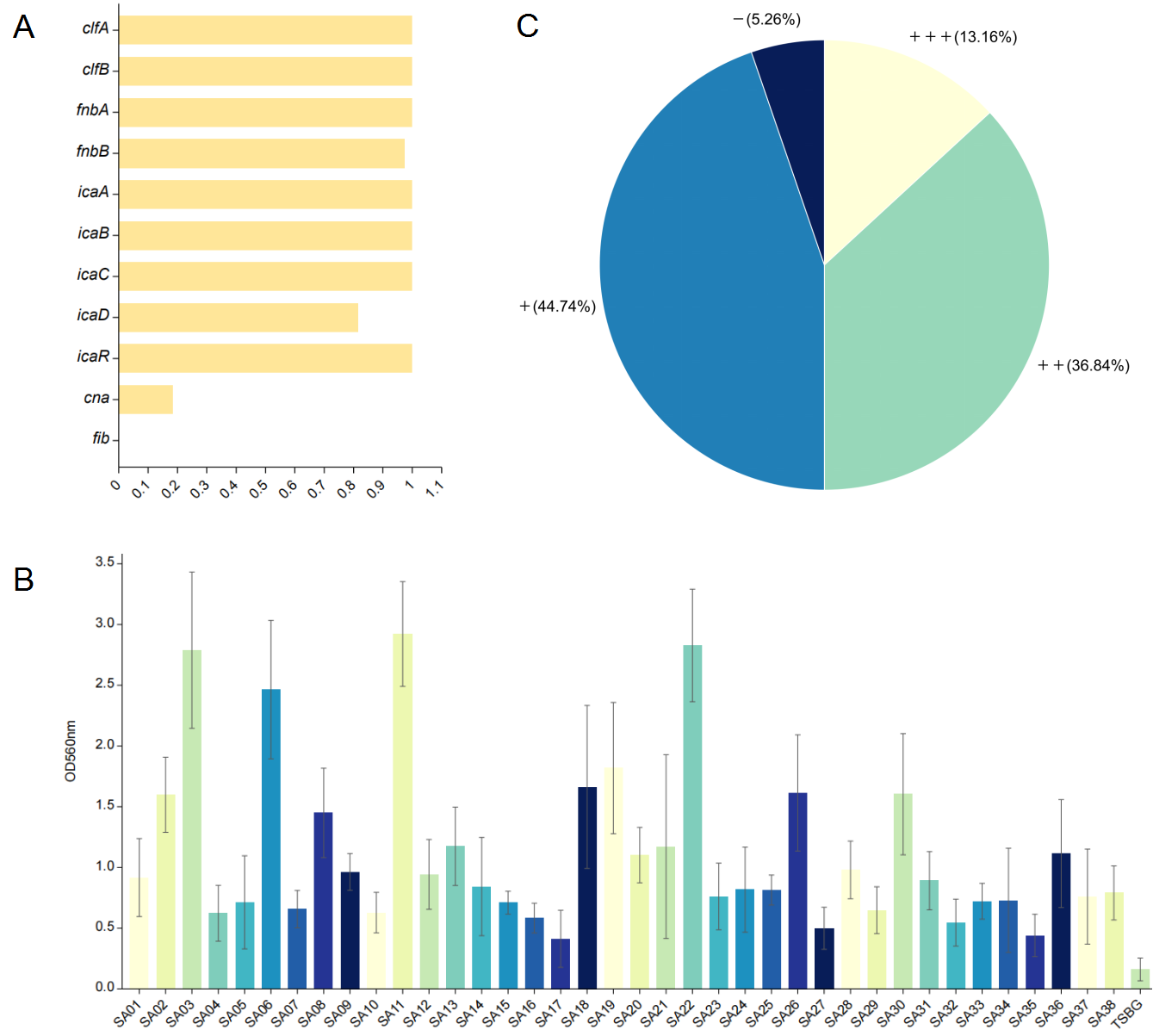


**Figure S1.** The biofilm formation of MRSA strains. **(A)** The detection rate of biofilm genes in MRSA strains. **(B)** The detection of biofilm formation ability of MRSA strains. **(C)** The proportion of biofilm formation ability.

**Table S1** Primer sequences and amplification conditions of all genes.

| Gene | Forward primer Reverse primer | Amplification conditions | Size/bp |
| --- | --- | --- | --- |
| *norA* | TTCACCAAGCCATCAAAAAG CTTGCCTTTCTCCAGCAATA | (94℃, 4min) + [(94℃, 30 s) + (60℃, 50 s) + (72℃, 55s)] × 30+ (72℃, 5 min) | 620 |
| *norB* | AGCGCGTTGTCTATCTTTCC GCAGGTGGTCTTGCTGATAA | (94℃, 4min) + [(94℃, 30 s) + (62℃, 50 s) + (72℃, 55s)] × 30+(72℃, 5 min) | 213 |
| *norC* | AATGGGTTCTAAGCGACCAA ATACCTGAAGCAACGCCAAC | (94℃, 4min) + [(94℃, 30 s) + (62℃, 50 s) + (72℃, 55s)] × 30+(72℃, 5 min) | 216 |
| *sepA* | GCAGTCGAGCATTTAATGGA ACGTTGTTGCAACTGTGTAAGA | (94℃, 4min) + [(94℃, 30 s) + (61℃, 50 s) + (72℃, 55s)] × 30+(72℃, 5 min) | 103 |
| *mepA* | ATGTTGCTGCTGCTCTGTTC TCAACTGTCAAACGATCACG | (94℃, 4min) + [(94℃, 30 s) + (61℃, 50 s) + (72℃, 55s)] × 30+(72℃, 5 min) | 718 |
| *mdeA* | AACGCGATACCAACCATTC TTAGCACCAGCTATTGGACCT | (94℃, 4min) + [(94℃, 30 s) + (61℃, 50 s) + (72℃, 55s)] × 30+(72℃, 5 min) | 677 |
| *qacA/B* | GCTGCATTTATGACAATGTTTG AATCCCACCTACTAAAGCAG | (95℃, 60s) + [(95℃, 60s) + (58℃, 45s) + (72℃, 60s)] × 30+(72℃, 5 min) | 628 |
| *smr* | ATAAGTACTGAAGTTATTGGAAGT TTCCGAAAATGTTTAACGAAACTA | (95℃, 60s) + [(95℃, 60s) + (58℃, 45s) + (72℃, 60s)] × 30+(72℃, 5 min) | 285 |
| *mecA* | AAAATCGATGGTAAAGGTTGGC AGTTCTGCAGTACCGGATTTGC | (95℃, 4min) + [(94℃, 60s) + (50℃, 30s) + (72℃, 1.5min)] ×30 + (72℃, 10 min) | 533 |
| *mecC* | GAAAAAAAGGCTTAGAACGCCTC GAAGATCTTTTCCGTTTTCAGC | (94℃, 5min) + [(94℃, 30s) + (59℃, 60s) + (72℃, 60s)] ×30 + (72℃, 10 min) | 138 |
| *aacA-aphD* | TGATAATGCCACAAATGTTAAGG CTCCAAAATCAATTATTCCAGT | (94℃, 5min) + [(95℃, 30s) + (50.7℃, 30s) + (72℃, 3min)] ×30 + (72℃, 5min) | 647 |
| *blaZ* | TGTAATTCAAACAGTTCACATGCC TCATTACACTCTTGGCGGTTT | (94℃, 5min) + [(94℃, 45s) + (57℃, 50s) + (72℃, 60s)] ×30+(72℃, 10min) | 787 |
| *ermA* | GTTCAAGAACAATCAATACAGAG GGATCAGGAAAAGGACATTTTAC | (94℃, 5min) + [(94℃, 30s) + (52℃, 30s) + (72℃, 60s)] ×30 + (72℃, 5min) | 421 |
| *ermB* | CCGTTTACGAAATTGGAACAGGTAAAGGGC GAATCGAGACTTGAGTGTGC | (94℃, 5min) + [(94℃, 30s) + (55℃, 30s) + (72℃, 60s)] ×30 + (72℃, 5min) | 359 |
| *ermC* | GCTAATATTGTTTAAATCGTCAATTCC GGATCAGGAAAAGGACATTTTAC | (94℃, 5min) + [(94℃, 30s) + (52℃, 30s) + (72℃, 60s)] ×30 + (72℃, 5min) | 572 |
| *fexA* | TTGGGAAGAATGGTTCAGGG ATCGGCTCAGTAGCATCACG | (94℃, 5min) + [(94℃, 30s) + (55.5℃, 30s) + (72℃, 60s)] ×32 + (72℃, 10min) | 977 |
| *gyrA* | TTACCAGTGAAATGCGTGAA TCGTTGCCATACCTACCG | (94℃, 5min) + [(94℃, 30s) + (51℃, 45s) + (72℃, 60s)] ×30 + (72℃, 10min) | 504 |
| *grlA* | CATTGCCAGATGTTCGT ACCACCAGTTGGAAAATC | (94℃, 5min) + [(94℃, 30s) + (49℃, 45s) + (72℃, 60s)] ×30 + (72℃, 10min) | 557 |
| *tet (L)* | GTTGCGCGCTATATTCCAAA TTAAGCAAACTCATTCCAGC | (94℃, 5min) + [(94℃, 30s) + (56℃, 30s) + (72℃, 60s)] ×30+(72℃, 10min) | 788 |
| *tet (k)* | TTAGGTGAAGGGTTAGGTCC GCAAACTCATTCCAGAAGCA | (94℃, 5min) + [(94℃, 30s) + (58℃, 30s) + (72℃, 60s)] ×30 + (72℃, 10min) | 718 |
| *tet (M)* | ACAGAAAGCTTATTATATAAC TGGCGTGTCTATGATGTTCAC | (94℃, 5 min) + [(94℃, 30 s) + (55℃, 30 s) + (72℃, 1 min)] ×30 + (72℃, 5 min) | 171 |
| *tet (O)* | ACGGARAGTTTATTGTATACC TGGCGTATCTATAATGTTGAC | (94℃, 5 min) + [(94℃, 30 s) + (60℃, 30 s) + (72℃, 1 min)] ×30 + (72℃, 5 min) | 171 |
| *lin (A)* | GGTGGCTGGGGGGTAGATGTATTAACTGG GCTTCTTTTGAAATACATGGTATTTTTCGATC | (94℃, 5 min) + [(94℃, 30 s) + (57℃, 30 s) + (72℃, 1 min)] ×32 + (72℃, 10 min) | 323 |
| *optrA* | AGGTGGTCAGCGAACTAA ATCAACTGTTCCCATTCA | (94℃, 5 min) + [(94℃, 45 s) + (47℃, 45 s) + (72℃, 1 min)] ×30 + (72℃, 10 min) | 1395 |
| *cfr* | TGAAGTATAAAGCAGGTTGGGAGTCA ACCATATAATTGACCACAAGCAGC | (94℃, 5 min) + [(94℃, 45 s) + (48℃, 45 s) + (72℃, 45 s)] ×30 + (72℃, 10 min) | 746 |
| *pvl* | ATCATTAGGTAAAATGTCTGGACATGATCCA GCATCAAGTGTATTGGATAGCAAAAGC | (93℃, 2min) + [(93℃, 60s) + (55℃, 60s) + (72℃, 60s)] ×35+(72℃, 30s) | 433 |
| *tst* | ACCCCTGTTCCCTTATCATC TTTTCAGTATTTGTAACGCC | (95℃, 4min) + [(95℃, 60s) + (55℃, 60s) + (72℃, 60s)] ×30+(72℃, 10min) | 326 |
| *eta* | ATATCAACGTGAGGGCTCTAGTAC ATGCAGTCAGCTTCTTACTGCTA | (95℃, 4min) + [(95℃, 30s) + (52℃, 30s) + (72℃, 1.5min)] ×25+(72℃, 5min) | 1155 |
| *etb* | CACACATTACGGATAATGCAAG TCAACCGAATAGAGTGAACTTATCT | (95℃, 4min) + [(95℃, 30s) + (52℃, 30s) + (72℃, 1.5min)] ×25+(72℃, 5min) | 604 |
| *sea* | CCTTTGGAAACGGTTAAAACG TCTGAACCTTCCCATCAAAAAC | (95℃, 4min) + [(95℃, 60s) + (55℃, 60s) + (72℃, 2min)] ×30+(72℃, 7min) | 127 |
| *seb* | TCGCATCAAACTGACAAACG GCAGGTACTCTATAAGTGCCTGC | (94℃, 5 min) + [(95℃, 60 s) + (55℃, 60 s) + (72℃, 60 s)] ×30+(72℃, 10 min) | 477 |
| *sec* | ACCAGACCCTATGCCAGATG TCCCATTATCAAAGTGGTTTCC | (94℃, 5 min) + [(94℃, 60 s) + (56℃, 60 s) + (68℃, 60 s)] ×30+(72℃, 7 min) | 371 |
| *sed* | CTGAATTAAGTAGTACCGCGCT TCCTTTTGCAAATAGCGCCTTG | (94℃, 10 min) + [(94℃, 30 s) + (55℃, 30 s) + (72℃, 30 s)] ×30+(72℃, 10 min) | 451 |
| *see* | CGGGGGTGTAACATTACATGAT CCCTTGAGCATCAAACAAATCATAA | (94℃, 10 min) + [(94℃, 30 s) + (55℃, 30 s) + (72℃, 30 s)] ×30+(72℃, 10 min) | 286 |
| *seg* | ATGTCTCCACCTGTTGAAGG TGAGCCAGTGTCTTGCTTTG | (94℃, 3 min) + [(94℃, 30 s) + (60℃, 30 s) + (72℃, 30 s)] ×30+(72℃, 10 min) | 400 |
| *seh* | TCACATCATATGCGAAAGCAG TCGGACAATATTTTTCTGATCTTT | (94℃, 5 min) + [(94℃, 60 s) + (56℃, 60 s) + (68℃, 60 s)] ×30+(72℃, 7 min) | 463 |
| *sei* | CTCAAGGTGATATTGGTGTAGG AAAAAACTTACAGGCAGTCCATTTC | (95℃, 5 min) + [(94℃, 60 s) + (55℃, 60 s) + (72℃, 60 s)] ×30+(72℃, 10 min) | 576 |
| *selj* | CAGCGATAGCAAAAATGAAACA TCTAGCGGAACAACAGTTCTGA | (94℃, 3 min) + [(94℃, 30 s) + (60℃, 30 s) + (72℃, 30 s)] ×30+(72℃, 10 min) | 426 |
| *selk* | GTGTCTCTAATAATGCCAGCGCT CGTTAGTAGCTGTGACTCCACC | (94℃, 10 min) + [(94℃, 30 s) + (55℃, 30 s) + (72℃, 30 s)] ×30+(72℃, 10 min) | 282 |
| *sell* | CACCAGAATCACACCGCTTA CTGTTTGATGCTTGCCATTG | (94℃, 5 min) + [(94℃, 60 s) + (56℃, 60 s) + (68℃, 60 s)] ×30+(72℃, 7 min) | 240 |
| *selm* | CTATTAATCTTTGGGTTAATGGAGAAC TTCAGTTTCGACAGTTTTGTTGTCAT | (94℃, 5min) +｛(94℃, 40s)+(58℃, 40s) + (72℃, 1.5min)]×30(The annealing temperature decreases by 0.5℃ for each cycle)+(72℃, 10min) | 300 |
| *seln* | TCATGCTTATACGGAGGAGTTACG AACCTTCTTGTTGGACACCATC | (94℃, 5min) +｛(94℃, 40s)+(58℃, 40s)+(72℃, 1.5min)]×30(The annealing temperature decreases by 0.5℃ for each cycle)+(72℃, 10min) | 103 |
| *selo* | AGTTTGTGTAAGAAGTCAAGTGTAGA ATCTTTAAATTCAGCAGATATTCCATCTAAC | (94℃, 5min) +｛(94℃, 40s)+(58℃, 40s)+(72℃, 1.5min)]×30(The annealing temperature decreases by 0.5℃ for each cycle)+(72℃, 10min) | 180 |
| *selp* | GAATTGCAGGGAACTGCTTT ACCAACCGAATCACCAGAAG | (94℃, 5min) +｛(94℃, 40s)+(58℃, 40s)+(72℃, 1.5min)]×30(The annealing temperature decreases by 0.5℃ for each cycle)+(72℃, 10min) | 537 |
| *selq* | GGTGGAATTACGTTGGCGAATCA CTCTGCTTGACCAGTTCCGGTG | (94℃, 5min) +｛(94℃, 40s)+(58℃, 40s)+(72℃, 1.5min)]×30(The annealing temperature decreases by 0.5℃ for each cycle)+(72℃, 10min) | 330 |
| *ser* | TTCAGTAAGTGCTAAACCAGATCC CTGTGGAGTGCATTGTAACGCC | (94℃, 5min) +｛(94℃, 40s)+(58℃, 40s)+(72℃, 1.5min)]×30(The annealing temperature decreases by 0.5℃ for each cycle)+(72℃, 10min) | 368 |
| *selu* | ATGGCTCTAAAATTGATGGTTCTA GCCAGTCTCATAAGGCGAACTA | (94℃, 5min) +｛(94℃, 40s)+(58℃, 40s)+(72℃, 1.5min)]×30(The annealing temperature decreases by 0.5℃ for each cycle)+(72℃, 10min) | 410 |
| *clfA* | CGCCGGTAACTGGTGAAGCT TGCTCTCATTCTAGGCGCACTT | (95℃, 5min) + [(95℃, 30 s) + (55℃, 30 s) + (72℃, 45 s)] ×30+(72℃, 10 min) | 314 |
| *clfB* | ATGATCTTGCTTGCGTT CCGATTCAAGAGTTACACC | (95℃, 5min) + [(95℃, 30 s) + (47℃, 30 s) + (72℃, 45 s)] ×30+(72℃, 10 min) | 215 |
| *fnbA* | GCGGAGATCAAAGACAA CCATCTATAGCTGTGTGG | (95℃, 5min) + [(95℃, 30 s) + (48℃, 30 s) + (72℃, 45 s)] ×35+(72℃, 10 min) | 1279 |
| *fnbB* | GGAGAAGGAATTAAGGCG GCCGTCGCCTTGAGCGT | (95℃, 5min) + [(95℃, 30 s) + (56℃, 30 s) + (72℃, 45 s)] ×35+(72℃, 10 min) | 820 |
| *fib* | CGTCAACAGCAGATGCGAGCG TGCATCAGTTTTCGCTGCTGGTTT | (94℃, 5min) + [(95℃, 30 s) + (54℃, 30 s) + (72℃, 3min)] ×35+(72℃, 10 min) | 239 |
| *cna* | AATAGAGGCGCCACGACCGTATAC GTGCCTTCCCAAACCTTTTGAGCA | (94℃, 5min) + [(95℃, 30 s) + (54℃, 30 s) + (72℃, 3min)] ×35+(72℃, 10 min) | 155 |
| *icaA* | CTTGCTGGCGCAGTCAATAC CCAACATCCAACACATGGCA | (94℃, 5min) + [(95℃, 30 s) + (54℃, 30 s) + (72℃, 3min)] ×35+(72℃, 10 min) | 178 |
| *icaC* | CTTGGGTATTTGCACGCATT GCAATATCATGCCGACACCT | (94℃, 5min) + [(95℃, 30 s) + (54℃, 30 s) + (72℃, 3min)] ×35+(72℃, 10 min) | 209 |
| *icaD* | CGCTATATCGTGTGTCTTTTGGA TCGCGAAAATGCCCATAGTT | (94℃, 5min) + [(95℃, 30 s) + (54℃, 30 s) + (72℃, 3min)] ×35+(72℃, 10 min) | 164 |
| *hla* | GTACTACAGATATTGGAAGC GTAATCAGATATTTGAGCTAC | (95℃, 5min) + [(95℃, 30 s) + (47℃, 30 s) + (72℃, 45 s)] ×38+(72℃, 10 min) | 274 |
| *hlb* | GCCAAAGCCGAATCTAAG CGCATATACATCCCATGGC | (95℃, 5min) + [(95℃, 30 s) + (51℃, 30 s) + (72℃, 45 s)] ×35+(72℃, 10 min) | 840 |

**Table S2** The basic information of 38 MRSA strains.

| ID | MLST | SCC*mec* | *spa* | Sex | Age | Department | Specimen type | Biofilm formation |
| --- | --- | --- | --- | --- | --- | --- | --- | --- |
| SA01 | ST59 | Ⅳ | t441 | Male | 5 years old | Otolaryngology | Purulent fluid | ＋＋ |
| SA02 | ST59 | Ⅴ | t437 | Male | 2 days old | Neonate | Secretion | ＋＋ |
| SA03 | ST254 | NT | t8 | Female | 38 years old | Colorectal department | Purulent fluid | ＋＋＋ |
| SA04 | ST398 | NT | t34 | Female | 29 years old | General surgery department | Purulent fluid | ＋ |
| SA05 | ST5 | NT | t548 | Female | 3 years old | Pediatrics | Purulent fluid | ＋ |
| SA06 | ST630 | NT | t337 | Male | 10 days old | Neonate | Secretion | ＋＋＋ |
| SA07 | ST1232 | Ⅴ | t34 | Male | 55 years old | Orthopaedics | Secretion | ＋ |
| SA08 | ST25 | NT | t3033 | Male | 9 years old | Pediatrics | Secretion | ＋＋ |
| SA09 | ST59 | Ⅳ | t437 | Female | 1 years old | Neonate | Secretion | ＋＋ |
| SA10 | ST59 | Ⅴ | t437 | Female | 15 days old | Otolaryngology | Secretion | ＋ |
| SA11 | ST121 | NT | t2019 | Female | 4 years old | Pediatrics | Secretion | ＋＋＋ |
| SA12 | ST25 | NT | t78 | Male | 78 years old | Neurosurgery | Purulent fluid | ＋＋ |
| SA13 | ST59 | Ⅳ | t437 | Female | 66 years old | Otolaryngology | Purulent fluid | ＋＋ |
| SA14 | ST59 | Ⅳ | t437 | Male | 45 years old | Endocrine | Purulent fluid | ＋ |
| SA15 | ST3685 | NT | t84 | Female | 2 months old | Neonate | Secretion | ＋ |
| SA16 | ST59 | NT | t437 | Male | 11 months old | Otolaryngology | Purulent fluid | ＋ |
| SA17 | ST22 | NT | t309 | Male | 6 years old | General surgery department | Purulent fluid | － |
| SA18 | ST59 | Ⅴ | t437 | Male | 4 years old | Otolaryngology | Purulent fluid | ＋＋ |
| SA19 | ST59 | Ⅳ | t437 | Female | 32 years old | Obstetrics | Vaginal swab | ＋＋＋ |
| SA20 | ST22 | Ⅴ | t309 | Male | 63 years old | Otolaryngology | Purulent fluid | ＋＋ |
| SA21 | ST59 | Ⅳ | t437 | Male | 32 years old | Orthopaedics | Secretion | ＋＋ |
| SA22 | ST3355 | Ⅳ | t437 | Female | 17 years old | Gynaecology | Tissue | ＋＋＋ |
| SA23 | ST59 | Ⅳ | t437 | Male | 3 days old | Neonate | Secretion | ＋ |
| SA24 | ST3355 | Ⅳ | t437 | Female | 23 years old | Otolaryngology | Pharyngeal swab | ＋ |
| SA25 | ST59 | Ⅳ | t441 | Male | 70 years old | ICU | Secretion | ＋ |
| SA26 | ST9 | Ⅻ | t899 | Male | 58 years old | Burn department | Secretion | ＋＋ |
| SA27 | ST88 | Ⅳ | NT | Male | 52 years old | Orthopaedics | Secretion | ＋ |
| SA28 | ST22 | NT | t309 | Female | 2 months old | Neonate | Secretion | ＋＋ |
| SA29 | ST1 | Ⅳ | t127 | Male | 3 years old | Pediatrics | Secretion | ＋ |
| SA30 | ST59 | Ⅳ | t441 | Male | 8 years old | Pediatrics | Secretion | ＋＋ |
| SA31 | ST5 | NT | t2 | Male | 10 days old | Neonate | Secretion | ＋＋ |
| SA32 | ST59 | Ⅳ | t437 | Female | 51 years old | Gynaecology | Secretion | ＋ |
| SA33 | ST59 | Ⅴ | t437 | Male | 48 years old | Burn department | Secretion | ＋ |
| SA34 | ST59 | Ⅴ | t437 | Male | 48 years old | Burn department | Secretion | ＋ |
| SA35 | ST5 | NT | t2 | Male | 9 days old | Neonate | Secretion | － |
| SA36 | ST59 | Ⅳ | t172 | Female | 73 years old | Intrarespiratory | Sputum | ＋＋ |
| SA37 | ST59 | Ⅴ | t437 | Female | 66 years old | Endocrine | Secretion | ＋ |
| SA38 | ST59 | Ⅳ | t437 | Male | 7 days old | Neonate | Secretion | ＋ |

**Table S3** The basic information of reference strains.

| ID | Name | Year | Continent | Source | Accession number |
| --- | --- | --- | --- | --- | --- |
| 1 | 79796 | 2017 | Asia | Human | PDT001646743.1 |
| 2 | SA957 | 2000 | Asia | Human | GCA_000470845.1 |
| 3 | SA40 | 2005 | Asia | Human | GCA_000470865.1 |
| 4 | AR3 | 2016 | Asia | Human | GCA_003310335.1 |
| 5 | M1 | 2014 | Asia | Human | GCA_001412275.1 |
| 6 | L16 | 2017 | Asia | Human | WLCC00000000 |
| 7 | 917-0 | 2012 | Asia | Food | WLBZ00000000 |
| 8 | 24-1 | 2013 | Asia | Food | WLAY00000000 |
| 9 | 3295 | 2015 | Asia | Food | WLAC00000000 |
| 10 | 3939A1 | 2016 | Asia | Food | WLBL00000000 |
| 11 | 4090A1 | 2016 | Asia | Food | WLBO00000000 |
| 12 | CQY3C006P | 2013 | Asia | Animal | GCA_011007235.1 |
| 13 | CQR3P007P | 2013 | Asia | Animal | GCA_011007265.1 |
| 14 | GDB8P68A | 2018 | Asia | Animal | GCA_018682235.1 |
| 15 | YK046 | 2015 | Asia | Animal | GCA_003309045.1 |
| 16 | M013 | 2002 | Asia | Human | GCA_000237125.3 |
| 17 | C11224 | 2006 | America | Human | GCA_028554155.1 |
| 18 | USA1000 | 2005 | America | Human | GCA_009799525.1 |
| 19 | SWF.27 | 2015 | Eurpoe | Human | PDT001600684.1 |
| 20 | CM28 | 2014 | Europe | Human | GCA_003240245.1 |
| 21 | M3386D | 2004 | Europe | Food | GCA_006511635.1 |
| 22 | W25799 | 2012 | America | Human | GCA_000603225.1 |
